# Gallic acid prevents isoproterenol-induced cardiac hypertrophy and fibrosis through regulation of JNK2 signaling and Smad3 binding activity

**DOI:** 10.1038/srep34790

**Published:** 2016-10-05

**Authors:** Yuhee Ryu, Li Jin, Hae Jin Kee, Zhe Hao Piao, Jae Yeong Cho, Gwi Ran Kim, Sin Young Choi, Ming Quan Lin, Myung Ho Jeong

**Affiliations:** 1 Heart Research Center of Chonnam National University Hospital, Gwangju 501-757, Republic of Korea; 2 Jilin Hospital Affiliated with Jilin University, Jilin, China; 3 The Second Hospital of Jilin University, Changchun, China; 4 Yanbian University Hospital, 1327 Juzi Road, Yanbian, Jilin 133000, China

## Abstract

Gallic acid, a type of phenolic acid, has been shown to have beneficial effects in inflammation, vascular calcification, and metabolic diseases. The present study was aimed at determining the effect and regulatory mechanism of gallic acid in cardiac hypertrophy and fibrosis. Cardiac hypertrophy was induced by isoproterenol (ISP) in mice and primary neonatal cardiomyocytes. Gallic acid pretreatment attenuated concentric cardiac hypertrophy. It downregulated the expression of atrial natriuretic peptide, brain natriuretic peptide, and beta-myosin heavy chain *in vivo* and *in vitro*. Moreover, it prevented interstitial collagen deposition and expression of fibrosis-associated genes. Upregulation of collagen type I by Smad3 overexpression was observed in cardiac myoblast H9c2 cells but not in cardiac fibroblasts. Gallic acid reduced the DNA binding activity of phosphorylated Smad3 in Smad binding sites of collagen type I promoter in rat cardiac fibroblasts. Furthermore, it decreased the ISP-induced phosphorylation of c-Jun N-terminal kinase (JNK) and extracellular signal regulated kinase (ERK) protein in mice. JNK2 overexpression reduced collagen type I and Smad3 expression as well as GATA4 expression in H9c2 cells and cardiac fibroblasts. Gallic acid might be a novel therapeutic agent for the prevention of cardiac hypertrophy and fibrosis by regulating the JNK2 and Smad3 signaling pathway.

Cardiac hypertrophy is regarded as a compensation mechanism to overcome the increased workload when the stress or injury is transient. Myocardial hypertrophy is characterized by increased heart mass, protein synthesis rate, sarcomeric reorganization, and activation of fetal genes such as atrial natriuretic peptide (ANP), brain natriuretic peptide (BNP), β-myosin heavy chain (β-MHC), and skeletal α-actin^1^. However, if the cardiac stress persists for a long time, the compensatory state can lead to a maladaptive condition called cardiac hypertrophy, which causes sudden death, arrhythmia, and heart failure^2^. Cardiac hypertrophy usually accompanies fibrosis, which is characterized by accumulation of extracellular matrix proteins such as collagen type I and fibronectin^3^,^4^.

Cardiac hypertrophy is a clinical hallmark of hypertrophic cardiomyopathy (HCM). Dilated cardiomyopathy (DCM) is type of HCM and involves myocardial fibrosis. To treat cardiac hypertrophy, fibrosis, and heart failure, angiotensin-converting enzyme inhibitors (ACEI), angiotensin receptor blockers (ARB), β-blockers, protein kinase C/D (PKC/PKD) inhibitors, calcium-calmodulin-dependent kinase (CaMKII) inhibitors, statins, and histone deacetylase (HDAC) inhibitors have been investigated as a therapeutic options^5^,^6^,^7^. A small pilot study has reported that spironolactone, a mineralocorticoid receptor antagonist, attenuates myocardial fibrosis in idiopathic DCM patients^8^. Another clinical study showed that losartan, an angiotensin II receptor antagonist, decreases myocardial collagen content in hypertensive patients with left ventricular hypertrophy^9^. However, there is insufficient clinical evidence or inadequate therapy for treatment of cardiac hypertrophy with fibrosis.

Transforming growth factor-β1 (TGF-β1)-Smad3 is a well-known signaling pathway in the process of fibrosis. Smad2 and Smad3 are downstream mediators of TGF-β1. Both Smad2 and Smad3 are phosphorylated by activated type I receptors, form a complex with Smad4, and translocate from cytoplasm to the nucleus. Smad2 and Smad3 counteract each other in fibrosis. For instance, Smad2 protects against fibrosis^10^, whereas Smad3 stimulates it^11^,^12^. Smad3-knockout mice showed attenuated cardiac fibrosis in response to hypertensive or diabetic cardiac hypertrophy^13^. Smads consist of Mad-homology 1(MH1), a linker, and MH2. The cross-talk between mitogen-activated protein kinase (MAPK) signaling and TGF-β1-Smad3 axis has been reported. For example, c-Jun N-terminal kinase (JNK) or p38 MAPK phosphorylates the intermediate linker of Smad3^14^,^15^,^16^.

Recent studies have shown that phytochemicals including flavonoid, quercetin, curcumin, and resveratrol ameliorate left ventricular hypertrophy *in vivo* and *in vitro*^17^,^18^,^19^,^20^. In addition, our group has reported that pretreatment with sulforaphane or piceatannol prevents cardiac hypertrophy through regulation of transcription factor GATA4 or GATA6^21^,^22^. These evidences indicate the potential of phytochemicals as therapeutics for pathological diseases. Gallic acid is one of phytochemicals. Gallic acid (3,4,5-trihydroxybenzoic acid) is found in many plants and products such as tea leaves, grapes, blackberry, and gallnuts. It is known to have anti-cancer, anti-oxidant, anti-microbial, anti-hyperglycemic, and anti-inflammatory activities^23^. Several reports have shown that gallic acid possesses protective effect against cardiovascular disorders, vascular calcification, and radiation^24^,^25^,^26^,^27^. The most recent data indicated that gallic acid regulates metabolic diseases^28^. However, the effect of gallic acid in cardiac hypertrophy and fibrosis has not been elucidated.

In the present study, we investigated the effects and the underlying mechanism of gallic acid in cardiac hypertrophy and fibrosis *in vivo* and *in vitro*. We clearly demonstrated that administration of gallic acid prevents cardiac hypertrophy and fibrosis through the regulation of the MAPK signaling pathway and Smad3-mediated collagen type I expression.

## Methods

### Reagents and antibodies

Isoproterenol (ISP), gallic acid (G7384), and sarcomere α-actinin (A7811) antibody were purchased from Sigma-Aldrich Co. (St. Louis, MO, USA). Anti-GAPDH (sc-32233), anti-BNP (sc-271185), anti-α smooth muscle actin (sc-130617), anti-ERK (sc-271269), and JNK (sc-7345) were from Santa Cruz Biotechnology (Dallas, Texas, USA); antibody to atrial natriuretic peptide (anti-ANP) was from Meridian Life Science (Memphis, TN, USA). Anti-β-MHC (ab50967), anti-collagen type I (ab34710), and anti-GATA4 (ab84593) antibodies were purchased from Abcam (Cambridge, MA, USA); anti-fibronectin (MA5-11981) was purchased from Thermo Fisher Scientific (Waltham, MA, USA). Anti-Smad3 (#9523), anti-phospho-Smad3 (#9520), anti-phospho-ERK1/2 (#437094), and anti-phospho-JNK1/2 (#9251) antibodies were purchased from Cell Signaling Technology (Danvers, MA, USA).

### Animals

Male CD-1(ICR) mice were purchased from the Orient Bio (South Korea). All animal experiments were approved by the Animal Experiment Committee of the Chonnam National University Medical School (CNU IACUC-H-2015-52) and carried out in accordance with the Guide for the Care and Use of Laboratory Animals (US National Institutes of Health Publication, 8^th^ edition, 2011).

### Animal model of cardiac hypertrophy

Animal experiments were divided into 2 different conditions. First, cardiac hypertrophy was induced by ISP infusion in mice for 2 weeks. The mice were pretreated with intraperitoneal gallic acid (100 mg/kg/day) 1 week before ISP infusion and co-administered ISP for additional 2 weeks. Mice were divided into four groups: vehicle-treated controls (n = 8), gallic acid-treated groups (n = 8), ISP-treated groups (n = 8), and ISP-treated groups with gallic acid pretreatment (n = 8).

Second, cardiac hypertrophy was induced by infusion of ISP in mice for 3 days. The mice were pretreated with gallic acid 2 weeks before ISP infusion and co-administered ISP for additional 3 days (n = 11~16 per group). ISP was dissolved using 0.1% ascorbic acid in 0.9% saline and was subcutaneously infused in mice at 25 mg/kg/day with an osmotic mini pump (Alzet) under ketamine and xylazine anesthesia (70 and 14 mg/kg, intraperitoneal injection, respectively).

### Left ventricle function by echocardiography

Transthoracic echocardiography was performed using a 13-MHz linear array transducer system (Vivid S5, GE, USA). Mice were anesthetized with ketamine (70 mg/kg) and xylazine (14 mg/kg) and the chest hair was shaved. The transducer was placed on the left hemithorax. Two dimensionally guided left ventricle (LV) M-mode images at the papillary muscle level were obtained from the parasternal short axis view. Left ventricular posterior thickness, interventricular septal thicknesses, and LV internal dimensions at end of diastole and systole were measured from M-mode images^29^. Fractional shortening (FS) of LV was calculated as follows: LV FS (%) = [(LVDd-LVSd)/LVDd] × 100, where LVDd is LV dimension at end-diastole and LVSd is LV dimension at end-systole.

### Blood pressure measurement

Blood pressure was examined as described previously^30^. Systolic blood pressures were measured 2 week after infusion of ISP by the tail-cuff method (Visitech Systems, BP-2000).

### Histological analysis

CD-1 mice were euthanized using a 100% grade CO_2_ for approximately 2–3 min. Immunohistochemistry was performed as described previously. Briefly, heart tissues were fixed with 4% paraformaldehyde, embedded in paraffin, and sectioned (3-μm- thick). For immunohistochemistry, the hearts were permeabilized in 0.1% Triton X-100 and antigen retrieval was performed. After blocking with 1% bovine serum albumin (BSA), heart sections were incubated with anti-collagen type I (1:400) overnight at 4 °C and treated with secondary antibody (Alexa Fluor 568 goat anti-rabbit IgG) for 1 h. Sudan black B solutions were used to reduce autofluorescence. Slides were mounted using ProLong Gold anti-fade reagent and stained with DAPI; images were observed under a fluorescence microscope (Nikon, Tokyo, Japan).

For determination of heart structure, heart tissues were incubated with Gill’s hematoxylin V for 3 min and washed with tap water. Slides were dipped 5~7 times in 0.25% HCl solution and then washed and dipped in 1% ammonium water 6~8 times. After dipping in 95% ethanol, tissues were incubated with Eosin Y for 2 min 30 sec. Tissues were dehydrated and mounted. Cross-section area was measured using ImageScope (300~350 areas/group). For assessment of size of cardiomyocytes from heart tissues, heart sections were prepared as mentioned above and incubated with wheat germ agglutinin conjugate of Alexa Fluor 488 (1:400) for 1 hour at room temperature. The cell size was measured using NIS Elements Software (Nicon, Japan).

### Masson’s trichrome staining

Cross-sections of whole hearts were stained with Masson’s trichrome to identify interstitial or perivascular collagen deposition. The black color and red color show nuclei and myocardium, respectively, while collagen fibers stained blue. Briefly, heart sections were deparaffinized. Tissues were incubated with Bouin’s solution at 56 °C for 30 min and washed with tap water. Slides were incubated with Weigert’s iron hematoxylin solution for 5 min, rinsed with running tap water, stained with Biebrich scarlet-acid fuchsin solution, and washed and incubated in phosphomolybdic-phosphotungstic acid solution. The slides were incubated with aniline Blue solution, treated with 1% acetic acid solution, dehydrated, and mounted with mounting solution.

### Cell culture

Primary rat neonatal cardiomyocytes were prepared from the hearts of 2-day-old Sprague-Dawley rats (Orient Bio, South Korea) as described previously^22^. Briefly, after euthanasia by decapitation, heart tissues were cut and digested with enzymatic solution (collagenase type II and pancreatin). The cells were isolated using Percoll step gradients. To remove cardiac fibroblasts, cells of Percoll layers were pre-plated for 40 min. The supernatant cells were seeded on gelatin-coated culture dishes. Cells were maintained in Dulbecco’s modified Eagle’s medium (DMEM) containing 10% fetal bovine serum (FBS) and 5-bromodeoxyuridine.

Fibroblasts attached to the cell culture dishes were maintained in DMEM with 10% FBS and used between passages 1 and 4. Isolation of primary cardiomyocytes and fibroblasts was at least 4 independently performed. H9c2 cardiomyoblast cells were obtained from the Seoul Korean Cell Line Bank (Seoul, Korea) and were maintained in DMEM containing 10% FBS. Cells were serum-starved overnight and pretreated with gallic acid or vehicle for 30 min and incubated with ISP for 24 h.

### Cell size and sarcomeric actinin organization

Rat neonatal cardiomyocytes or H9c2 cells were plated on cover slips and fluorescent immunocytochemistry was performed as described previously^31^. After treatment with ISP or gallic acid, cells were fixed with paraformaldehyde, permeabilized with 0.2% Triton-X 100, and visualized by staining with sarcomeric α-actinin antibody (1:200, primary antibody) followed by secondary antibody (Alexa Fluor 568 goat anti-mouse IgG). DAPI was used to detect nuclei and the cell surface areas (52~61 per groups) were measured by use of NIS Elements Software (Nicon, Japan).

### Western blot analysis

Western blots were performed as described previously^32^. Cells or tissue lysates were incubated with RIPA buffer (150 mM NaCl, 1% Triton X-100, 1% sodium deoxycholate, 50 mM Tris-HCl, pH 7.5, 2 mM EDTA, 5 mM NaF, and 5 mM Na_3_VO_4_) containing protease inhibitors. Proteins were separated by 8~12% SDS-PAGE and then transferred to polyvinylidene difluoride (PVDF) membranes. The membranes were probed with the indicated antibodies and were developed using Immobilon Western Detection Reagents (Millipore, Billerica, MA, USA).

### Chromatin immunoprecipitation (ChIP) assay

ChIP assay was performed as described previously^30^. Briefly, primary rat cardiac fibroblasts were cross-linked with 1% formaldehyde for 10 min. The sonicated chromatin was immunoprecipitated with p-Smad3 or normal rabbit IgG antibody, and protein-DNA complexes were eluted. After reversing cross-links, DNA was purified and DNA amounts were analyzed by SYBR green PCR kit with specific regions of collagen type I promoter (−2357 to −2143). The PCR primers used in ChIP assay are presented in Supplementary Table 1.

### Real-time reverse transcription-polymerase chain reaction (RT-PCR)

Total RNA from heart tissue was isolated with TRIzol reagent (Invitrogen Life Technologies), and 1 μg of RNA was used for the reverse transcription reaction with TOPscript RT DryMIX (Enzynomics, South Korea). Quantification of mRNA levels was done with the SYBR Green PCR kit (Enzynomics, South Korea). The PCR primers used in this study are shown in Supplementary Table 1.

### Statistical analysis

Statistical analysis was performed using either Student’s *t*-test or one-way ANOVA with a Bonferroni post hoc test using software provided by GraphPad Prism version 5.0. Data are presented as means ± SD. A *P* value of <0.05 was considered statistically significant.

## Results

### Gallic acid pretreatment suppresses cardiomyocyte hypertrophy *in vitro*

**Figure 1:**
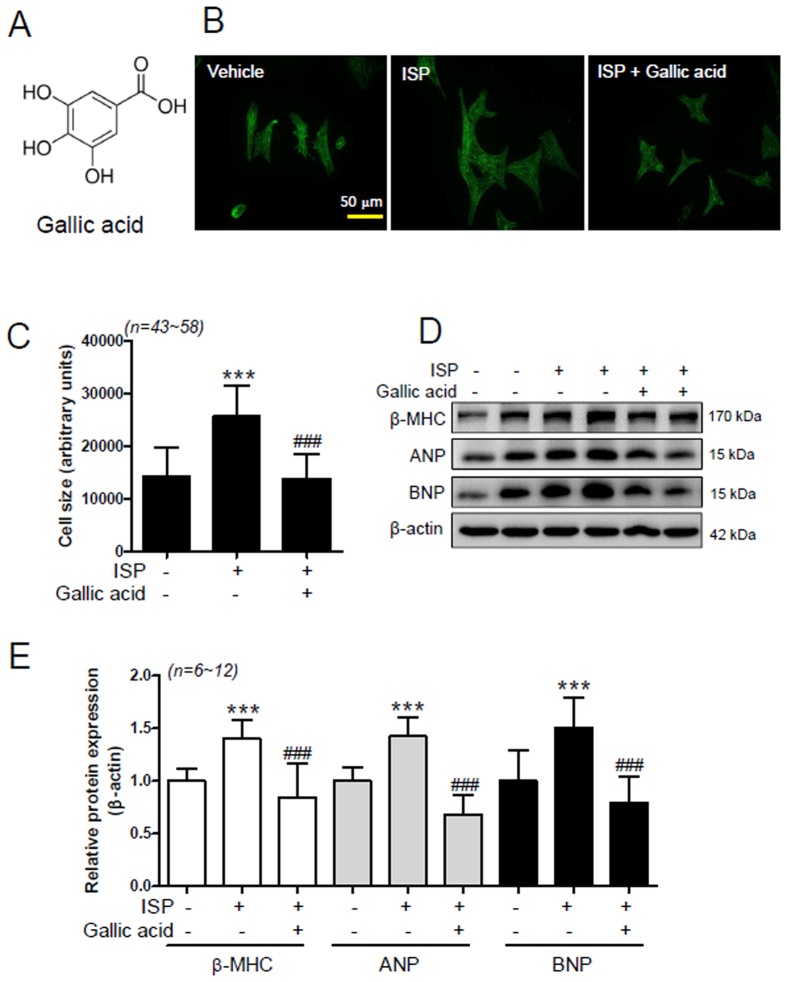
Gallic acid pretreatment suppresses cardiomyocyte hypertrophy *in vitro*. (**A**) Chemical structure of gallic acid. (**B**–**E**) Rat neonatal cardiomyocytes were pretreated with vehicle or 100 μmol/L gallic acid for 30 min and then coincubated with 10 μmol/L isoproterenol (ISP) for 12 h or 24 h. (**B**) Stress fiber was evaluated by immunocytochemistry with anti-sarcomeric α-actinin antibody (scale bar = 50 μm). (**C**) Cell size was calculated by measuring the cell surface area (n = 43~58 per group). Data are presented as the means ± SD of 3 independent experiments. ***P < 0.001 versus vehicle; ^###^*P* < 0.001 versus ISP + vehicle. (**D**) Cell lysates were subjected to SDS-PAGE and incubated with anti-β myosin heavy chain (β-MHC), atrial natriuretic peptide (ANP), and brain natriuretic peptide (BNP). β-Actin was used as a loading control. (**E**) β-MHC, ANP, and BNP protein amounts were quantified by densitometry. Data are presented as the means ± SD of 3 independent experiments. ***P < 0.001 versus vehicle; ^###^*P* < 0.001 versus ISP + vehicle.

To determine the effect of gallic acid on cardiomyocyte hypertrophy, we performed immunocytochemistry with anti-sarcomeric α-actinin antibody to evaluate cell size and sarcomere organization. As shown in Fig. 1A,B, gallic acid completely decreased the ISP (10 μmol/L)-induced increase in size of rat neonatal cardiomyocytes. We next examined the effects of gallic acid on expression of cardiac hypertrophic marker genes. Pretreatment with gallic acid significantly reduced the ISP-induced increase in β-MHC, ANP, and BNP expression (Fig. 1C–F) in rat neonatal cardiomyocytes. Similar results were observed in H9c2 cardiomyoblast cells (see Supplementary material, Fig. 1).

### Gallic acid prevents cardiac hypertrophy in ISP-treated mice

**Figure 2:**
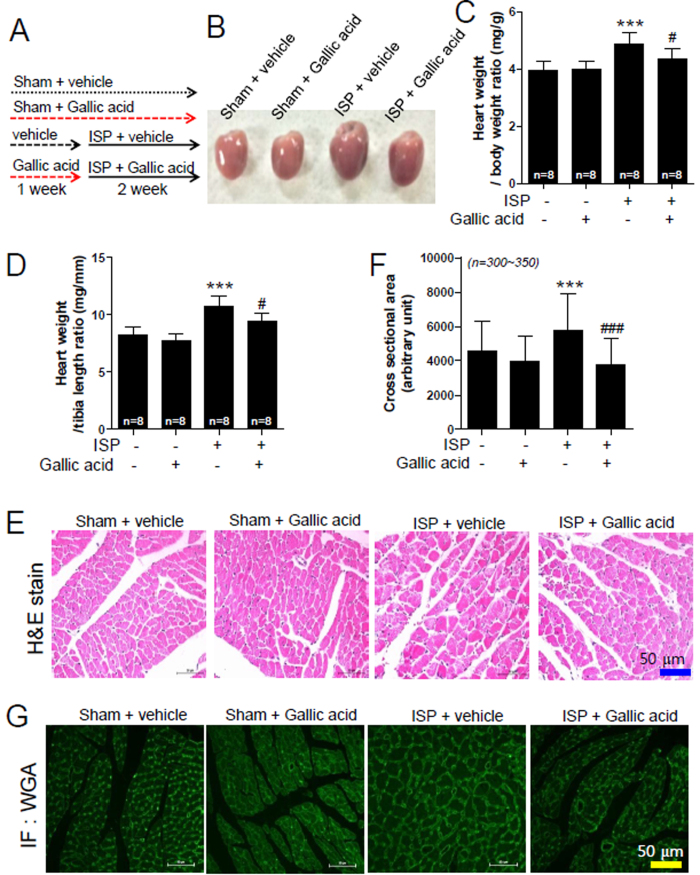
Gallic acid prevents cardiac hypertrophy in isoproterenol (ISP)-treated mice. (**A**) Schematic experimental protocol. Gallic acid administration was started 1 week before infusion of ISP (2 week) in mice; sham + vehicle, sham + gallic acid, ISP + vehicle, and ISP + gallic acid group. (**B**) Representative pictures of gross hearts. (**C**,**D**) Heart weight to body weight ratio and heart weight to tibia length ratio were analyzed 2 weeks after ISP infusion (n = 8 per group). (**E**) Representative H&E-stained images are shown (scale bar, 50 μm). ISP infusion indicates the enlarged myocardium. (**F**) Quantification of cross-sectional areas in H&E-stained hearts from 4 mice in each group (n = 300~350 per group). (**G**) Wheat germ agglutinin staining was performed to evaluate the cell size in heart sections (scale bar, 50 μm). Data are presented as the means ± SD. ***P < 0.001 versus sham; ^#^*P* < 0.05 and ^###^*P* < 0.001 versus ISP + vehicle.

To identify whether gallic acid has preventive effect on the development of cardiac hypertrophy, we pretreated mice with gallic acid for 1 week and then co-administered gallic acid and ISP using osmotic mini pump for 2 weeks (Fig. 2A). The dose of gallic acid used in animal experiments was determined based on the results of several studies showing suppressive effect on metabolic disorders^33^. As shown in Fig. 2B, ISP-induced cardiac enlargement was attenuated by pretreatment with gallic acid (100 mg/kg/day, intraperitoneal injection). This dose was selected based on a previous report^34^, and was found to be well tolerated by mice.

Pretreatment with gallic acid significantly decreased heart weight to body weight ratio (Fig. 2C) and heart weight to tibia length ratio (Fig. 2D). H&E staining showed that gallic acid completely reduced ISP-induced increase in cross-sectional area (Fig. 2E,F). We further observed that myocardial hypertrophy induced by ISP was attenuated by pretreatment with gallic acid, as demonstrated by wheat germ agglutinin staining (WGA, Fig. 2G). These data indicate that gallic acid pretreatment inhibited cardiac hypertrophy.

**Figure 3:**
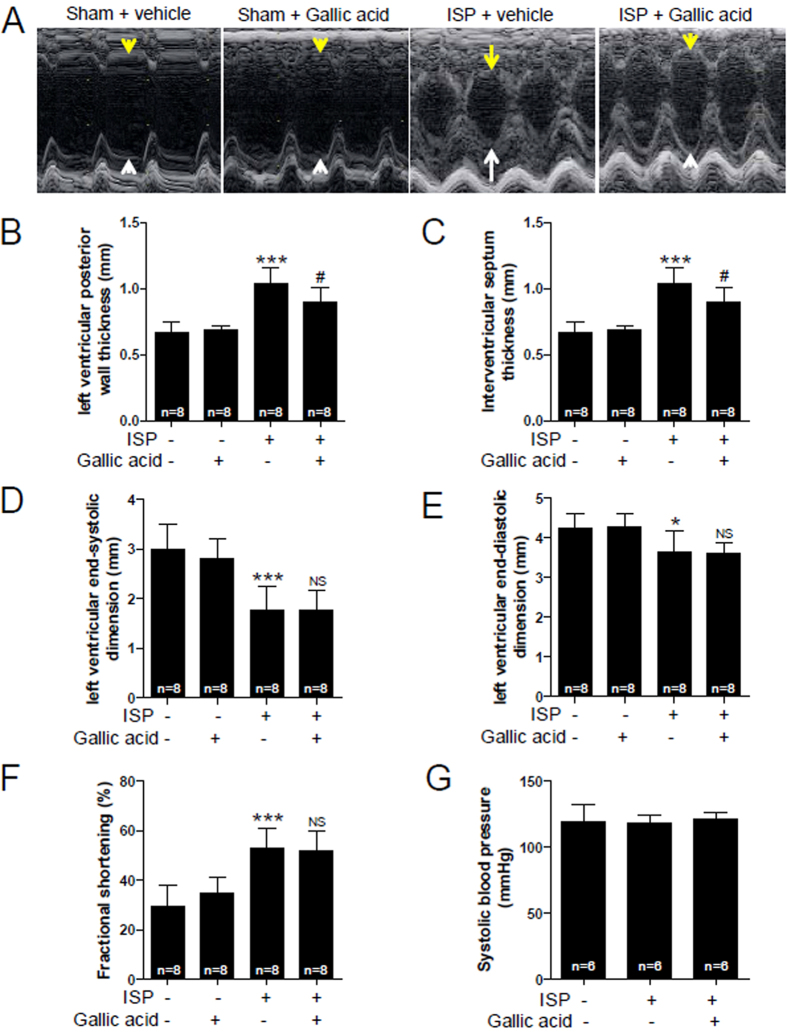
Gallic acid attenuates concentric cardiac hypertrophy in isoproterenol (ISP)-treated mice. (**A**) Representative M-mode echocardiograms are shown. Gallic acid administration was started 1 week before infusion of ISP in mice; sham + vehicle, sham + gallic acid, ISP + vehicle, and ISP + gallic acid group. White arrows and yellow arrows indicate the left ventricular posterior wall thickness and septum thickness, respectively. (**B**) Left ventricular posterior wall thickness (mm) (**C**) Interventricular septum thickness (mm). (**D**) Left ventricular end-systolic dimension (mm). (**E**) Left ventricular end-diastolic dimension (mm). (**F**) Fractional shortening (%). Data are presented as the means ± SD of 8 mice per group. (**G**) Systolic blood pressure was determined 2 weeks after ISP infusion. *P < 0.05 and ***P < 0.001 versus sham + vehicle; ^#^*P* < 0.05 versus ISP + vehicle. NS indicates not significant.

To further assess whether gallic acid affects left ventricular function, we performed echocardiography. Representative echocardiogram showed that the thickness of left ventricular septum and posterior wall was increased by 2-week ISP infusion and gallic acid pretreatment significantly decreased the thickness (Fig. 3A–C). ISP administration reduced left ventricular end-systolic dimension and end-diastolic dimension (Fig. 3D,E), indicating the induction of concentric hypertrophy in this mouse model. However, gallic acid pretreatment did not restore the reduced left ventricular end-systolic and diastolic dimension (Fig. 3D,E). Fractional shortening (%) is used to evaluate the left ventricular global systolic function. We found that fractional shortening increased in ISP-treated mice compared with the sham group (Fig. 3F). ISP did not affect systolic blood pressure (Fig. 3G). Taken together, these observations indicate that gallic acid pretreatment regulates left ventricle hypertrophy without causing left ventricle dysfunction or hypertension.

### Gallic acid suppresses the expression of genes involved in cardiac hypertrophy and left ventricular hypertrophy in mice treated with short-term infusion of isoproterenol (ISP)

**Figure 4:**
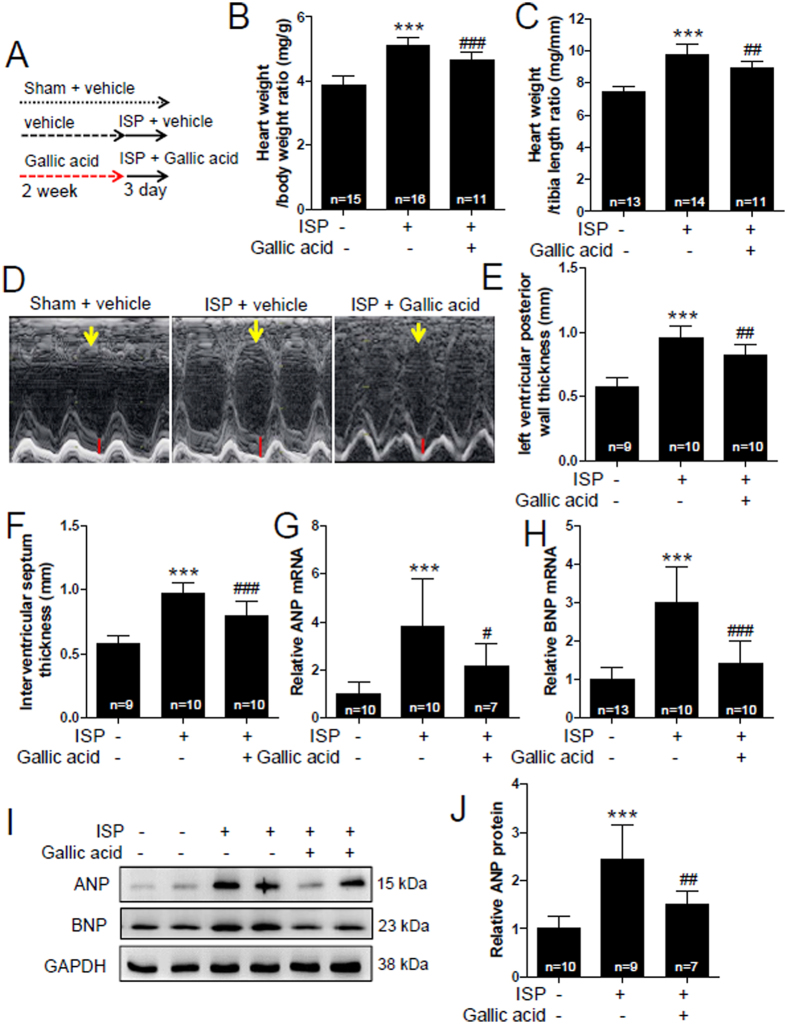
Gallic acid suppresses the expression of genes involved in cardiac hypertrophy and left ventricular wall thickness in mice treated with short-term infusion of isoproterenol (ISP). (**A**) Schematic experimental protocol. Gallic acid administration was started 2 weeks before infusion of ISP (3 days) in mice; sham + vehicle (n = 15), ISP + vehicle (n = 16), and ISP + gallic acid group (n = 11). (**B**) Heart weight to body weight ratio and (**C**) heart weight to tibia length ratio were assessed in mice. (**D**) Representative M-mode echocardiographic images after 3-day infusion of ISP in mice. Red lines and yellow arrows indicate the left ventricular posterior wall thickness and septum thickness, respectively. (**E**) Quantification of left ventricular posterior wall thickness (mm) and (**F**) interventricular septum thickness (mm) in ISP-induced mice. (**G**) ANP and (**H**) BNP mRNA was evaluated by quantitative real-time reverse transcription-polymerase chain reaction. (**I**) Representative immunoblots of ANP and BNP in mice heart. GAPDH was used as a loading control. (**J**) ANP protein was quantified by densitometry. Data are presented as the means ± SD. ***P < 0.001 versus sham + vehicle; ^#^*P* < 0.05, ^##^*P* < 0.01, and ^###^*P* < 0.001 versus ISP + vehicle. NS indicates not significant.

To identify whether short-term ISP administration is sufficient for the induction of cardiac hypertrophy, mice were infused with ISP for 3 days. Preliminary data showed that left ventricular septum and posterior thickness significantly increased at 3 day following ISP infusion (see Supplementary material, Fig. 2). In contrast, left ventricular end-systolic dimension decreased 3 days after ISP. Strikingly, heart weight to body weight ratio dramatically enhanced 2 days after ISP infusion (see Supplementary material, Fig. 2). We next investigated the effect of gallic acid on short-term ISP infusion-induced cardiac hypertrophy (Fig. 4A). As shown in Fig. 4B,C, pretreatment with gallic acid attenuated heart weight to body weight ratio and heart weight to tibia length ratio induced by ISP administration. Echocardiography demonstrated that gallic acid pretreatment ameliorated the ISP-induced increase in left ventricular posterior wall and septum thickness (Fig. 4D–F). Fetal gene activation is known as a feature of cardiac hypertrophy^35^. To investigate whether gallic acid can modulate fetal gene expression, we performed qRT-PCR using both the hearts. Both ANP and BNP mRNA and protein levels were increased by ISP infusion and this increase was reduced by gallic acid pretreatment (Fig. 4G–J).

### Gallic acid decreases cardiac fibrosis in mice treated with short-term isoproterenol (ISP) infusion

**Figure 5:**
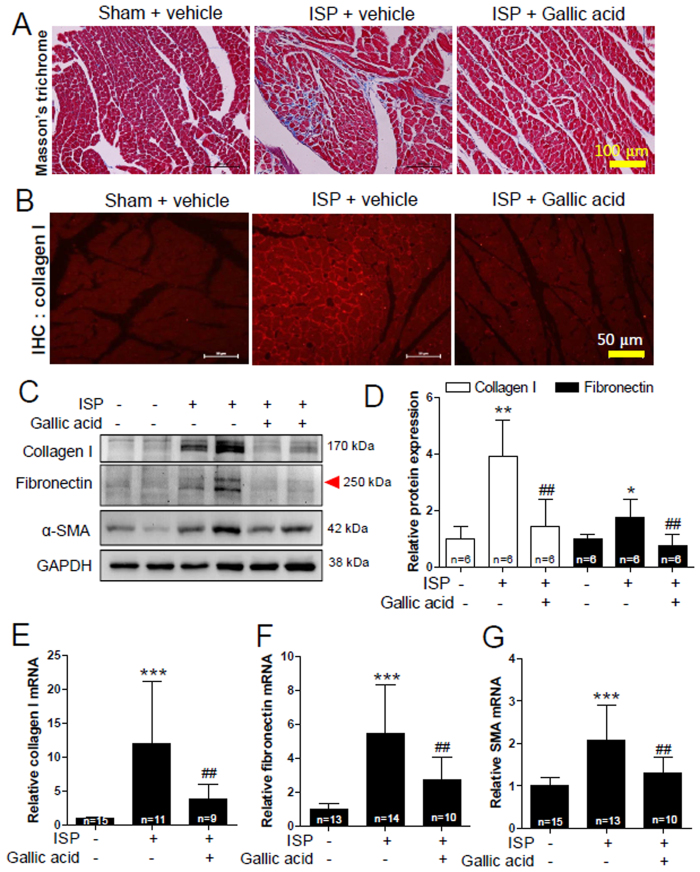
Gallic acid pretreatment suppressed cardiac fibrosis in isoproterenol (ISP)-treated mice. Gallic acid administration was started 2 weeks before infusion of ISP (3 days) in mice; sham + vehicle, ISP + vehicle, and ISP + gallic acid group. (**A**) Representative images of hearts stained with Masson’s trichrome, which were used to examine fibrosis (collagen deposition, scale bar, 100 μm). (**B**) Immunofluorescent staining with anti-collagen type I of heart sections from ISP-induced mice (scale bar, 50 μm). (**C**) Protein lysates from heart tissues were subjected to western blot with anti-collagen type I, anti-fibronectin, and anti-α-smooth muscle actin (SMA) antibodies. GAPDH was used as a loading control. (**D**) Collagen type I and fibronectin proteins were quantified by densitometry. (**E**–**G**) The mRNA levels of fibrosis-related genes (collagen type I, fibronectin, SMA) were analyzed by qRT-PCR. Data are presented as the means ± SD of 6~15 mice per group. *P < 0.05, *P < 0.01, and ***P < 0.001 versus sham + vehicle; ^##^*P* < 0.01versus ISP + vehicle.

Fibrosis, which is characterized by accumulation of extracellular matrix proteins, usually occurs in cardiac hypertrophy. We examined whether ISP induces cardiac fibrosis. As shown in Fig. 5A, collagen deposition increased in short-term ISP infusion-treated hearts compared to sham groups. This increase was dramatically decreased by gallic acid pretreatment. Immunohistochemistry (IHC) demonstrated that gallic acid pretreatment suppressed the increased collagen type I expression in heart sections from ISP-treated mice (Fig. 5B). We then investigated the effects of gallic acid on fibrosis-related gene expression. Gallic acid pretreatment reduced ISP-induced expression of collagen type I, fibronectin, and α-smooth muscle actin protein in mice (Fig. 5C,D). Consistent with these results, pretreatment with gallic acid significantly reduced mRNA levels of fibrosis-related genes (Fig. 5E–G).

### Gallic acid inhibits activated JNK2, which is involved in hypertrophy and fibrosis

**Figure 6:**
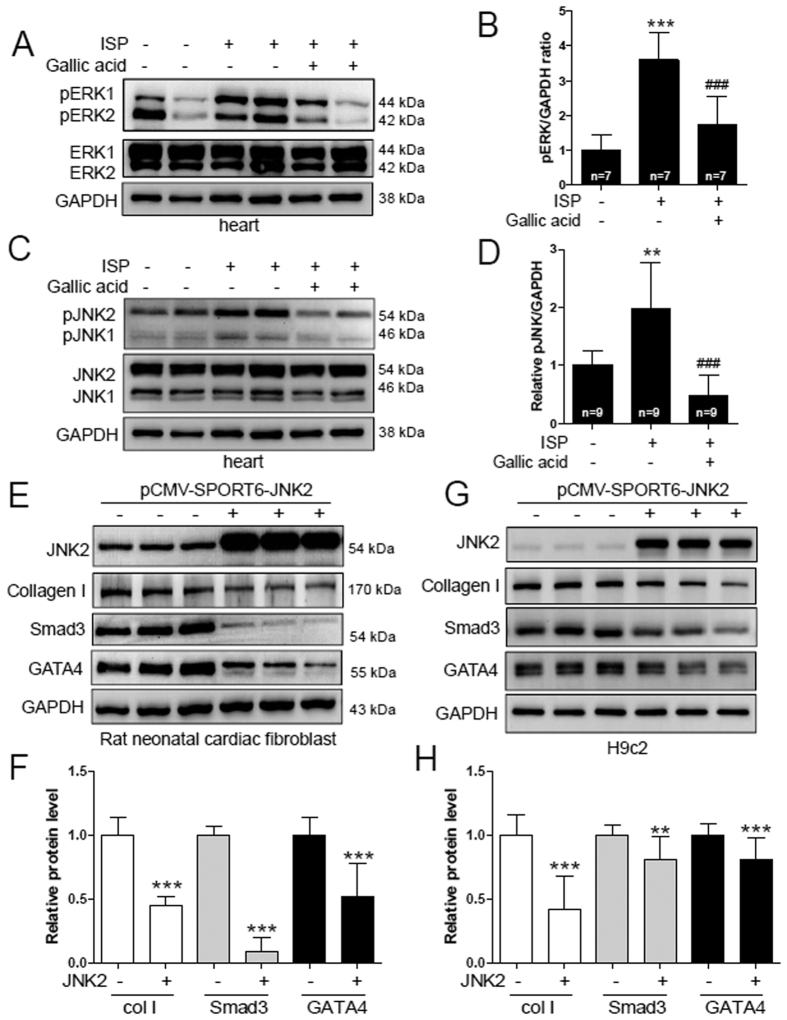
Gallic acid inhibits activated JNK2, which is involved in hypertrophy and fibrosis. Gallic acid administration was started 2 weeks before infusion of ISP (3 days) in mice; sham + vehicle, ISP + vehicle, and ISP + gallic acid group. (**A**,**C**) Representative images of immunoblots of the hearts of ISP mice. Proteins were subjected to western blotting with anti-p-ERK1/2, ERK1/2, p-JNK1/2, and JNK1/2. GAPDH was used as a loading control. (**B**,**D**) p-ERK and p-JNK proteins were quantified by densitometry. **P < 0.01 and ***P < 0.001 versus sham + vehicle; ^###^*P* < 0.001versus ISP + vehicle. (**E**) Rat neonatal cardiac fibroblasts and (**G**) H9c2 cells were transfected with pCMV-SPORT6-JNK2 or empty vector. Western blot results for JNK1/2, collagen type I, Smad3, and GATA4. GAPDH was used as a loading control. (**F**,**H**) Proteins were quantified by densitometry. Results are shown as means ± SD of 3 independent experiments. *P < 0.05. **P < 0.01, and ***P < 0.001 versus vector-transfected cells.

The MAPK signaling cascade has been reported to be involved in cardiac hypertrophy^36^. We investigated whether ISP induces the MAPK signaling pathway and whether gallic acid affects MAPK signaling. Pretreatment with gallic acid attenuated the ISP-induced increase in the phosphorylation levels of extracellular signal regulated kinase 1/2 (ERK1/2) in ISP-treated mice heart (Fig. 6A,B). Furthermore, ISP induced the phosphorylation of c-Jun N-terminal kinase 1/2 (JNK1/2) in mice, which was reduced by gallic acid pretreatment (Fig. 6C,D). However, p38 MAPK was unaffected by ISP (data not shown). We observed that p-JNK2 protein levels increased at 6 h following ISP treatment in rat cardiac fibroblasts (see Supplementary material, Fig. 3). However, p-ERK1/2 protein levels were unaffected. Therefore, we hypothesized that JNK2 could be involved in fibrosis. To identify the role of JNK2 in cardiac hypertrophy and fibrosis, rat neonatal cardiac fibroblasts and H9c2 cells were transfected with pCMV-SPORT6-JNK2 or empty vector. JNK2 overexpression inhibited GATA4 protein levels both in fibroblasts and in H9c2 cells (Fig. 6E–H). Furthermore, forced expression of JNK2 decreased Smad3 and collagen type I protein levels both in fibroblasts and in H9c2 cells (Fig. 6E–H).

### Gallic acid attenuates activated Smad3-mediated collagen type I expression in rat cardiac fibroblasts

**Figure 7:**
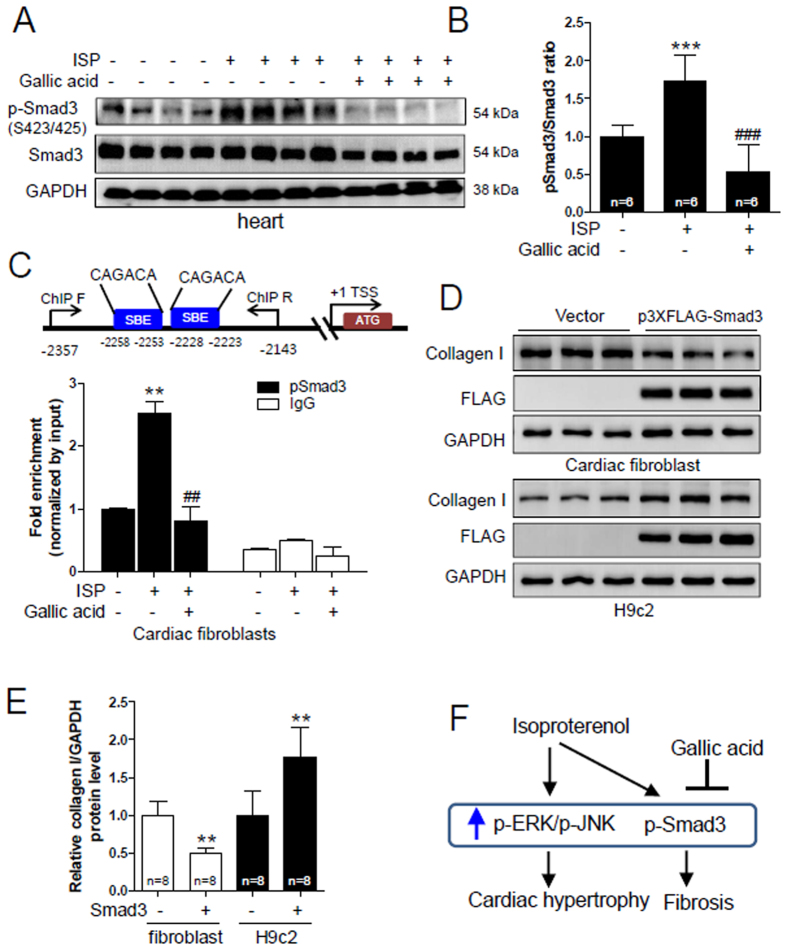
Gallic acid attenuates activated Smad3-mediated collagen type I expression in rat cardiac fibroblasts. Gallic acid administration was started 2 weeks before infusion of ISP (3 days) in mice; sham + vehicle, ISP + vehicle, and ISP + gallic acid group. (**A**) Representative images of immunoblots of the hearts of ISP mice. Proteins were subjected to western blotting with anti-p-Smad3 (S423/S425) and Smad3. GAPDH was used as a loading control. (**B**) p-Smad3 protein was quantified by densitometry. **P < 0.01 versus sham + vehicle; ^#^*P* < 0.05 versus ISP + vehicle. (**C**) Schematic diagram shows the putative Smad binding elements (SBE) in the rat collagen type I promoter. Putative two Smad3 binding elements (SBE, 5′-CAGACA-3′) are indicated in blue box. TSS indicates transcription start site. ChIP F and ChIP R indicate the PCR primer sets (−2357 to −2143) used for ChIP assay. Primary rat cardiac fibroblasts were treated with ISP in the absence or presence of gallic acid for 6 h. Chromatin DNA complexes were immunoprecipitated with anti-pSmad3 or anti-normal rabbit IgG antibody. Results are shown as means ± SD of 3 independent experiments. **P < 0.01 versus sham + vehicle; ^##^*P* < 0.01 versus ISP + vehicle. (**D**) Rat cardiac fibroblasts and H9c2 cells were transfected with p3 × FLAG-CMV-10-Smad3 or empty vector. Western blot analysis was performed. Collagen type I and FLAG expression was determined. (**E**) Collagen type I protein was quantified by densitometry (n = 8). **P < 0.01 and ***P < 0.001 versus vector-transfected cells. (**F**) Schematic diagram of gallic acid action on ISP-induced cardiac hypertrophy and fibrosis.

Smad3 is a well-known transcription factor in the process of fibrosis^37^. To determine whether Smad3 is associated with cardiac fibrosis, we performed western blot analysis. As shown in Fig. 7A,B, ISP-induced hearts showed an increase in p-Smad3 protein levels, which were reduced by gallic acid pretreatment. Furthermore, pretreatment with gallic acid reduced the Smad3 protein expression (Fig. 7A *and* see Supplementary material, Fig. 4). To delineate the inhibitory action of gallic acid on ISP-induced fibrosis, we performed a ChIP assay using primary rat neonatal cardiac fibroblasts. We confirmed that p-Smad3 protein expression increased from 3 h up to 12 h after ISP treatment in rat fibroblasts (see Supplementary material, Fig. 5). Therefore, fibroblasts were treated with gallic acid in the presence or absence of ISP for 6 h. Two Smad binding element (SBE) sites (−2258 to −2253/−2228 to −2223) were observed in the rat collagen type I promoter. SBE comprises the core sequence 5′-CAGACA-3′ (Fig. 7C*, upper panel*). ChIP assay showed that ISP increased the DNA binding activity of p-Smad3 in the rat collagen type I promoter, which was significantly diminished by gallic acid treatment (Fig. 7C*, lower panel*). We observed that Smad3 DNA binding was not altered in response to ISP stimulus (data not shown). We further investigated whether Smad3 directly regulates fibrosis-related gene expression both in rat cardiac fibroblasts and in H9c2 cells. Unexpectedly, Smad3 overexpression decreased the protein expression of collagen type I in cardiac fibroblasts (Fig. 7D,E), whereas, Smad3 overexpression significantly increased collagen type I protein levels in H9c2 cells (Fig. 7D,E).

## Discussion

The present study shows that gallic acid prevents cardiac hypertrophy and fibrosis both in isoproterenol-induced mouse model and in primary cardiac or fibroblast cells. First, the protective effect of gallic acid in cardiac hypertrophy is associated with the MAPK signaling pathway (Fig. 7F). Second, gallic acid suppresses the protein expression of phosphorylated Smad3 and Smad3, which are the major mediators of fibrosis. Third, gallic acid reduces the downstream target collagen type I of Smad3 (Fig. 5C) through regulation of Smad3 DNA binding activity (Fig. 7C). These results suggest that gallic acid is a novel therapeutic agent for amelioration of cardiac hypertrophy and fibrosis.

In this study, we for the first time demonstrated that pretreatment with gallic acid is protective against the cardiac hypertrophic stimulus. Indeed, gallic acid ameliorated ISP-induced cardiac enlargements as determined by examination of cross-sectional area and heart weight to body weight ratio, and echocardiography. Gallic acid is a natural compound and is regarded as relatively safe. This suggests that gallic acid could be used as a food supplement for preventing pathological heart diseases.

ISP, phenylephrine, endothelin-1, and angiotensin II induce cardiac hypertrophy^38^. Spontaneously hypertensive rats (SHR) are considered an essential model of hypertension accompanying cardiac hypertrophy. Angiotensin II induces hypertension and cardiac hypertrophy, whereas ISP induces cardiac hypertrophy, but not hypertension. Indeed, we observed that ISP did not increase systolic blood pressure (Fig. 3G). We assume that gallic acid did not act as antihypertensive agent in the absence of hypertensive stimuli.

In the present study, we showed that cardiac hypertrophy is induced by 2-week infusion of ISP as well as short-term administration of ISP (3 day) as evaluated by echocardiography. Furthermore, heart weight to body weight ratio significantly increased 2 days after infusion of ISP (see Supplementary material, Fig. 2G). Our findings suggest that mice receiving short-term infusion of ISP are a favorable animal model to evaluate the early stage of cardiac hypertrophy and fibrosis. Infusion of ISP (up to 2 weeks) induces concentric cardiac hypertrophy, which is characterized by an increase in wall thickness of left ventricles (LV) as well as a decrease in lumen of LV (Fig. 3). Indeed, ISP-induced mouse model is not an eccentric cardiac hypertrophic model, which responds to volume overload^39^. Unlike ISP-induced cardiac hypertrophy model, transverse aortic constriction (TAC) model exhibits an elevation in end-diastolic volume^40^. ISP-induced cardiac hypertrophy model has a limitation of increased fractional shortening, which is an estimate of myocardial contractility. Considering the fact that TAC model is a good animal model of pathological cardiac hypertrophy, future studies are needed to determine the effect of gallic acid in a pressure overload- induced hypertrophy model such as TAC model.

Activation of fetal genes such as β-MHC, ANP, and BNP is a hallmark of cardiac hypertrophy^41^. We demonstrated that gallic acid pretreatment reduces fetal gene program in cardiac hypertrophy induced by ISP in mice or cardiomyocytes (Figs 1C–F and 4G–J). Various hypertrophic stimuli activate receptors or channels and trigger several signaling cascades. The best-known signaling pathway is the MAPK pathway^42^. The MAPK cascades are divided into p38 kinases, JNKs^43^, and ERK1/2. The strong activation of ERK and JNK by ISP stimuli was consistent with the findings of previous studies^44^. Gallic acid pretreatment inhibited the activation of JNK1/2 and ERK1/2 *in vivo*. There are controversial results of JNK’s role in cardiac fibrosis. A study showed that deletion of JNK1 in the heart increased fibrosis following pressure overload^45^; conversely, apoptosis signal-regulating kinase 1 (ASK1)-JNK deficient mice showed attenuated cardiac fibrosis^46^. Furthermore, JNK has been reported to be activated by bile duct ligation or CCl4 administration in liver fibrosis. However, JNK1-deficient mice and JNK2-deficient mice displayed a reverse phenotype upon bile duct ligation^47^. While our data did not show the direct effect of JNK2 overexpression on fibrosis, forced expression of JNK2 decreased the expression of fibrosis-related genes such as collagen type I and Smad3 both in rat cardiac fibroblasts and in H9c2 cells (Fig. 6E–H). Further studies are needed to identify the exact role of JNK1/2 in the process of fibrosis.

In this study, we clearly showed that gallic acid pretreatment prevents cardiac fibrosis as determined by Masson’s trichrome staining and examination of fibrosis-related gene expression (Fig. 5). Indeed, gallic acid pretreatment inhibited collagen accumulation induced by ISP in mice and it downregulated the mRNA and protein levels of fibronectin, collagen type I, and α-smooth muscle actin. Adult mouse heart consists of cardiomyocytes (55%) and nonmyocytes (~27% fibroblasts) as evaluated by flow cytometric analysis^48^. Among several cell types, fibroblasts play a major role in stimulating fibrosis. The possible regulatory mechanism by which gallic acid inhibits fibrosis seems to involve Smad3 transcription factor. Our results provide a novel evidence of how gallic acid regulates Smad3 or collagen type I expression. Interestingly, activated Smad 3 seems to be involved in gallic acid-mediated reduction of collagen type I expression as demonstrated by the ChIP assay. Smad3 has been reported to be involved in fibrogenesis. However, our data showed Smad3 overexpression-mediated upregulation of collagen type I in H9c2 cells but not in cardiac fibroblasts. The discrepant results between two different cells were not explained in the study. Smad3-knockout mice did not show cardiac fibrosis after angiotensin II infusion^12^. Although TGF-β1/Smad3 signaling is closely involved in fibrosis^37^, the ISP/Smad3 signaling pathway seems to play a critical role in cardiac fibrosis. In the present study, gallic acid was found to reduce p-Smad3 protein levels more than total Smad3 protein levels in ISP-induced cardiac fibrosis. Considering the induction and the increased binding activity of the phosphorylated Smad3 in ISP-stimulated cardiac fibroblasts, the phosphorylation of Smad3 seems to play a more critical role than that of total Smad3 in cardiac fibrosis.

In summary, our studies have demonstrated that mice receiving short-term infusion of ISP are an effective model to induce cardiac hypertrophy and fibrosis. We showed that gallic acid prevents LV hypertrophy and fibrosis via inhibition of the JNK2 signaling pathway and Smad3 binding activity. Taken together, the results indicate that gallic acid may be a novel therapeutic agent for heart diseases involving cardiac fibrosis and hypertrophy.

## Additional Information

**How to cite this article**: Ryu, Y. *et al*. Gallic acid prevents isoproterenol-induced cardiac hypertrophy and fibrosis through regulation of JNK2 signaling and Smad3 binding activity. *Sci. Rep.* **6**, 34790; doi: 10.1038/srep34790 (2016).

## Supplementary Material

Supplementary Information
