# Supplementary material for: Gallic acid prevents isoproterenol-induced cardiac hypertrophy and fibrosis through regulation of JNK2 signaling and Smad3 binding activity
Source: Sci Rep. 2016 Oct 5;6:34790. doi: 10.1038/srep34790 (PMC5050511; doi:10.1038/srep34790)

Gallic acid prevents isoproterenol-induced cardiac hypertrophy and fibrosis through regulation of JNK2 signaling and Smad3 binding activity

Yuhee Ryu[1,+], Li Jin[1,2+], Hae Jin Kee[1,*], Zhe Hao Piao[3], Jae Yeong Cho[1], Gwi Ran Kim[1], Sin Young Choi[1], Ming Quan Lin[1,4], and Myung Ho Jeong[1,*]

[1]Heart Research Center of Chonnam National University Hospital, Gwangju 501-757, Republic of Korea.

[2]Jilin Hospital Affiliated with Jilin University, Jilin, China

[3]The Second Hospital of Jilin University, Changchun, China

[4]Yanbian University Hospital, 1327 Juzi Road, Yanbian, Jilin 133000, China

Supplementary Data

Supplementary Table 1

Supplementary Table 1. Primers for reverse transcription polymerase chain reaction (RT-PCR) and chromatin immunoprecipitation (ChIP)

Gene (mouse)	Primer sequence (5′ to 3′)
ANP	F: TGGAGGAGAAGATGCCGGTAGAAGAT<br>R: AGCGAGCAGAGCCCTCAGTTTGCT
BNP	F: CTGAAGGTGCTGTCCCAGAT<br>R: GTTCTTTTGTGAGGCCTTGG
Collagen I	F: GAGCGGAGAGTACTGGATCG<br>R: GCTTCTTTTCCTTGGGGTTC
Fibronectin	F: GATGCACCGATTGTCAACAG<br>R: TGATCAGCATGGACCACTTC
Alpha SMA	F: ACTGGGACGACATGGAAAAG<br>R: AGAGGCATAGAGGGACAGCA
GAPDH (mouse)	F: GCATGGCCTTCCGTGTTCCT<br>R: CCCTGTTGCTGTAGCCGTATTCAT
Collagen type I (ChIP)	F: TGAGAAGTGGCAGAGGAGGT<br>R: GACTGCCACATCAAGGGTCT

F, forward; R, reverse.

2. Supplementary Figures Legends

Supplementary Figure 1. Effect of gallic acid on isoproterenol (ISP)-treated H9c2 cells. H9c2 cells were incubated with ISP I (10 μmol/L) in the presence or absence of gallic acid (100 μmol/L). (*A*) Cells were fixed with 4% paraformaldehyde and incubated with sarcomeric α-actinin (1:200). Nuclei were stained with DAPI. Merged images are shown. (*B*) Cell size was measured. (*C*) Protein lysates were resolved using sodium dodecyl sulfate polyacrylamide gel electrophoresis (SDS-PAGE) and immunoblotted with the indicated antibodies. Representative western blots are shown. (*D-F*) Quantification of β-MHC, ANP, and BNP protein levels was performed using densitometry. Protein expression was normalized to GAPDH. Values are means ± SD of 3 independent experiments. $^{*}P < 0.05$ versus vehicle. $^{\#}P < 0.05$ and $^{\#\#}P < 0.01$ versus ISP.

Supplementary Figure 2. Echocardiographic parameters in ISP-infused mice. (*A*) Representative M-mode images of left ventricles in mice infused with ISP (1~3 day) as indicated. (*B–F*) Left ventricular posterior wall thickness, interventricular septum thickness, left ventricular end-systolic dimension, left ventricular end-systolic dimension, and fractional shortening were measured after ISP infusion. $^{*}P < 0.05$, $^{**}P < 0.01$, and $^{***}P < 0.001$ versus control. (*G*) Heart weight to body weight ratio.

Supplementary Figure 3. Expression of MAPK signaling in ISP-treated cardiac fibroblasts. Rat cardiac fibroblasts were serum-starved and incubated with ISP I (10 μmol/L) at indicated time. Proteins were analyzed by western blot with anti-p-ERK1/2 (Thr 202/Thy204), ERK, p-JNK1/2 (S423/425), and JNK antibodies. β-Actin was used as an internal control.

Supplementary Figure 4. Expression of Smad3 in ISP-treated cardiac fibroblasts. Gallic acid administration was started 2 week before infusion of ISP (3 day) in mice; sham + vehicle, ISP + vehicle, and ISP + gallic acid group. Proteins were subjected to western blotting with anti-Smad3 antibody. GAPDH was used as a loading control. Smad3 protein was quantified by densitometry. $^{\#}P<0.05$ versus ISP + vehicle. NS indicates not significant.

Supplementary Figure 5. Expression of phosphorylated Smad3 in ISP-treated cardiac fibroblasts. Rat cardiac fibroblasts were serum-starved and incubated with ISP I (10 μmol/L) at indicated time. Proteins were analyzed by western blot with anti-pSmad3 (S423/S425) and Smad3 antibodies. Glyceraldehyde 3-phosphate dehydrogenase (GAPDH) was used as an internal control.

Supplementary Figure 1

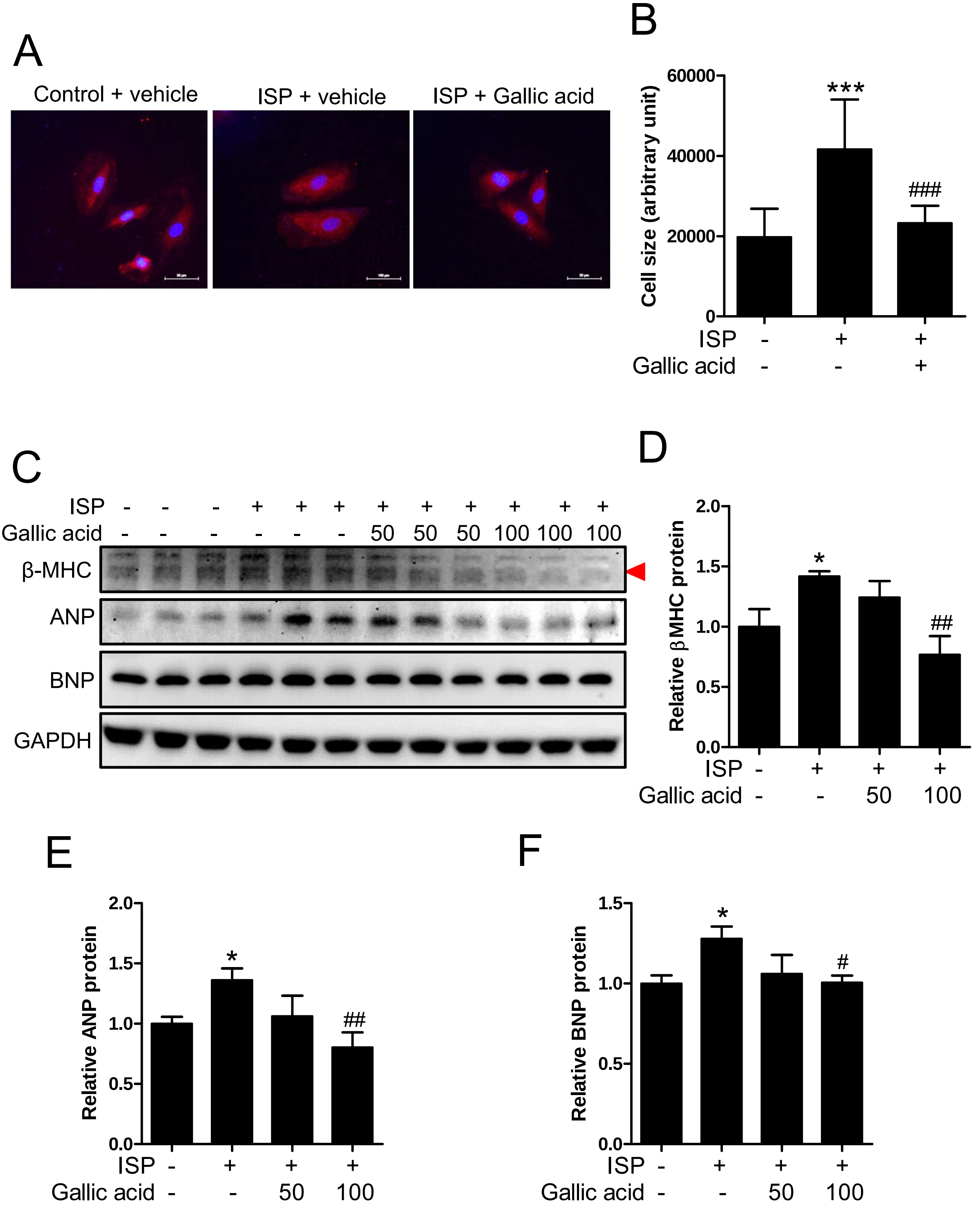

Supplementary Figure 2

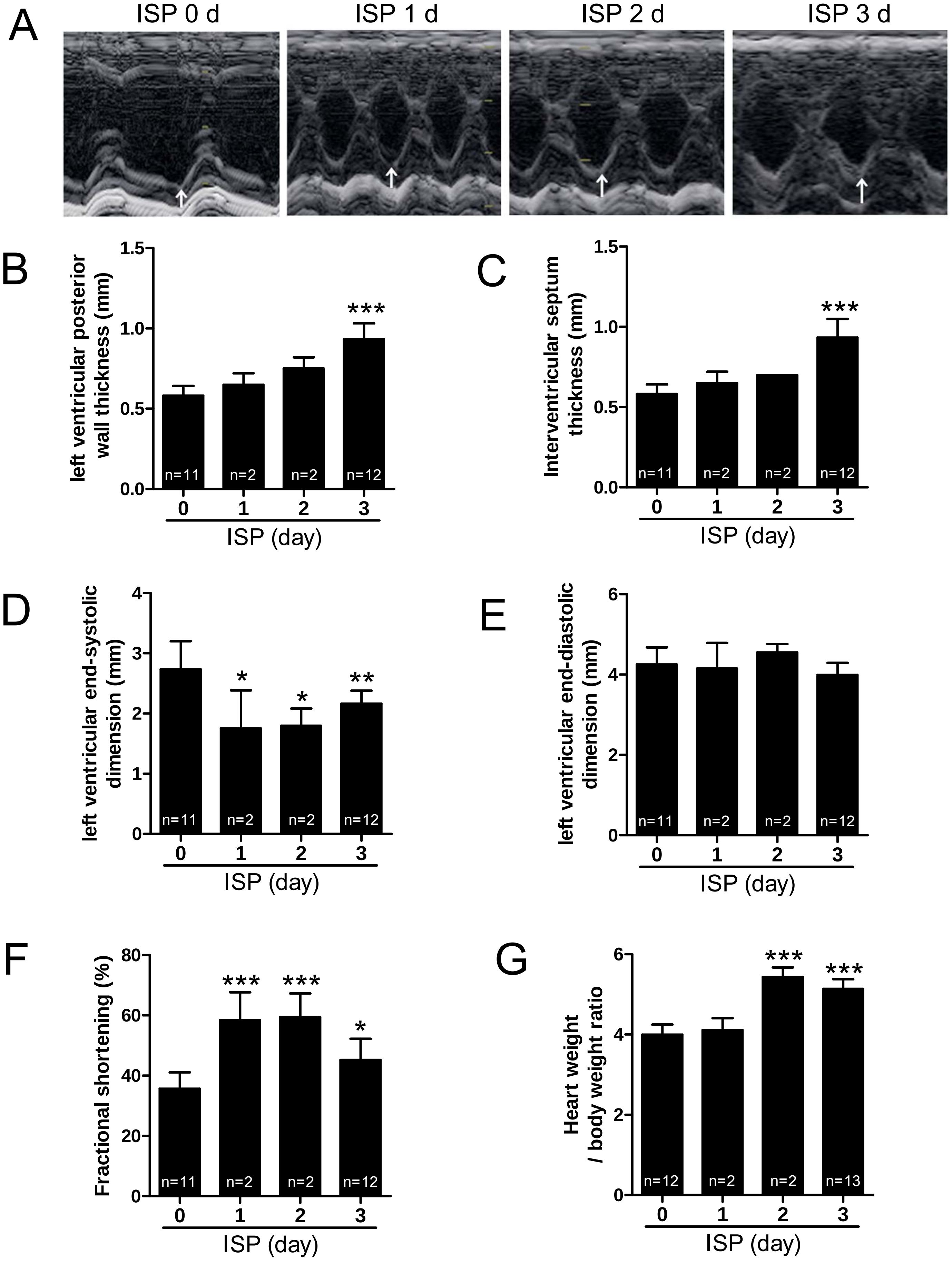

Supplementary Figure 3

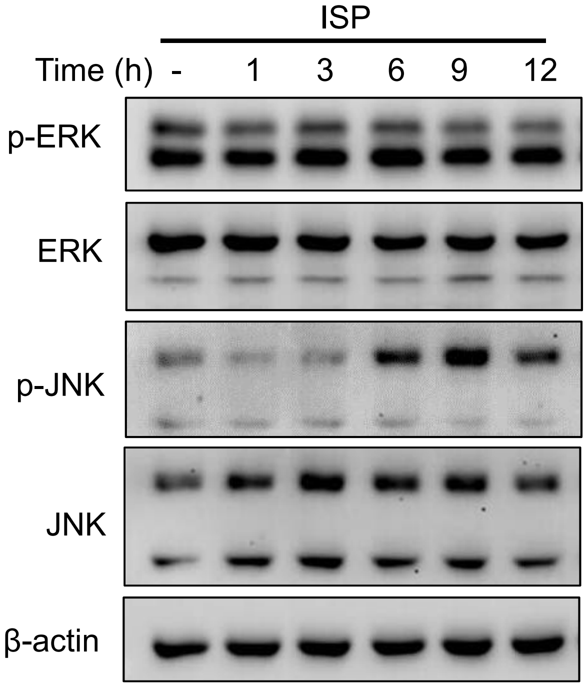

Rat cardiac fibroblast

Supplementary Figure 4

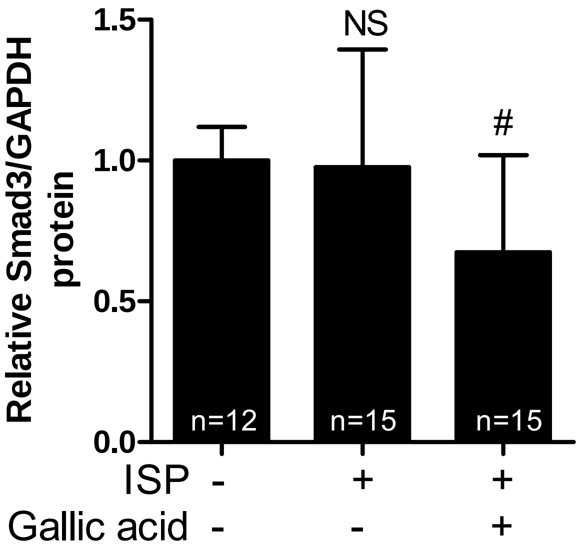

Supplementary Figure 5

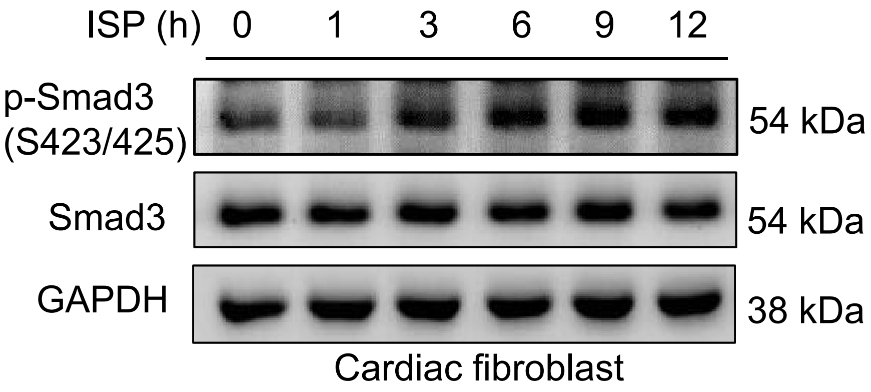

Supplement: Supplementary Information [file srep34790-s1.pdf]
